# The Ag–Li system's experimental and *ab initio* thermodynamic dataset

**DOI:** 10.1016/j.dib.2019.104939

**Published:** 2019-12-05

**Authors:** M. Helena Braga, Adam Dębski, Sylwia Terlicka, Wladyslaw Gąsior, Anna Góral

**Affiliations:** a LAETA, Engineering Physics Department, FEUP, University of Porto, R. Dr. Roberto Frias s/n, 4200-465 Porto, Portugal; b Institute of Metallurgy and Materials Science, Polish Academy of Sciences, 30-059 Kraków, 25, Reymonta Street, Poland

**Keywords:** Enthalpy of formation, Gibbs energy of formation, XRD, Vibrational heat capacity at constant volume, Thermal linear expansion coefficient, Phase diagrams

## Abstract

The Ag–Li system was analysed using first-principles calculations 10.1016/j.jallcom.2019.152811 [1]. The method included using density functional theory to optimize the crystal structure of the phases constituting the binary phase diagram by relaxing atomic positions, volume, and shape. The optimized structures were subsequently used to calculate thermodynamic properties at different temperatures; by determining the zero-point energy, the vibrational internal energy, and the entropy, the heat capacity at constant volume was obtained as well as the phases' stability limits. Furthermore, optimized structures were used to calculate the XRD patterns and to compare them with experimental data. All the referred data are now accessible to researchers and industrials demanding to work with binary and higher-order systems that include Ag and Li, for example, for energy storage. Binaries should be well assessed prior to higher-order phase diagrams and in that resides additional usefulness to this data.

**Table:** Specifications Table

Subject	Metals and Alloys
Specific subject area	Experimental and calculated Thermodynamic and structural data for the assessment of the Ag–Li system with applications in energy storage and high-temperature solders
Type of data	Table
How data were acquired	Instruments: Structural studies, D2 Phaser (Bruker, Cu Kα radiation) diffractometer Software: VASP, MT, and Phonon as implemented in Materials Design (2.22.6, 2019)
Data format	Raw
Parameters for data collection	The experimental structural XRD data were obtained using a CuKα radiation source. The calculated data were obtained using a plane wave cut-off of at least 400.00 eV and k-spacings of 0.230 × 0.230 × 0.230 Å^−1^
Description of data collection	The XRD data were obtained with a Bragg Brentano configuration for polycrystalline samples with a wavelength of λ = 0.1542 nm. The calculated data were obtained by building the crystal structure of the phase and optimizing it using VASP and allowing structure, volume, and atomic sites to relax and then, in a subsequent run, using MT or phonon to obtain the Thermodynamic properties vs temperature.
Data source location	Institution: University of Porto – FEUP City/Town/Region: Porto Country: Portugal Latitude and longitude (and GPS coordinates) for collected samples/data: Latitude: 41°10′45.59″ N Longitude: −8°35′40.74″ W
Data accessibility	With the article “Experimental and *ab initio* study of the Ag–Li system for energy storage and high-temperature solders” Mendeley DOI: https://doi.org/10.17632/vfpy3w6yn3.1
Related research article	Author's names: M. H. Braga, A. Dębski, S. Terlicka, W. Gąsior, A. Góral Title: Experimental and *ab initio* study of the Ag–Li system for energy storage and high-temperature solders Journal: JALCOM https://doi.org/10.1016/j.jallcom.2019.152811

**Table:** 

**Value of the Data** • These data are useful for the research and for the industry related to energy storage materials and high temperature solders • The crystallography and CALPHAD researchers will benefit from these data; the latter because they need experimental and *ab initio* data to assess binary and higher-order phase diagrams • The phase diagram needs to be reassessed and the experimental data are scarce since high reactivity of lithium at elevated temperature with the air contained elements (O_2_, N_2_, H_2_O) and the high energy effects accompanying the respective reactions makes it very difficult to obtain good quality results, therefore, the calculated data provides new insights for samples preparation and experimental planification

## 1 Data

1 **-** Ag30Li70-experimental-XRD.txt

X-ray diffraction pattern for the Ag_30_Li_70_ alloy, including settings on the experimental run, followed by two columns with the 2θ(°) and Intensity(a.u.) normalized to an I_max_ = 100. No zero-shift correction and no normalization were performed. The configuration of the diffractometer is Bragg-Brentano and the sample was polycrystalline. The source used was CuKα.

2 **-** Ag4Li9-gamma-disordered-calculated-XRD.txt

X-ray diffraction simulated pattern constituted by two columns with 2θ(°) and Intensity(a.u.) normalized to I_max_ = 100 for the γ-Ag_4_Li_9_ disordered phase. The simulated source used was CuKα.

3 **-** Ag3Li10_gamma_disordered_calculated-XRD.txt

X-ray diffraction simulated pattern constituted by two columns with 2θ(°) and Intensity(a.u.) normalized to I_max_ = 100 for the γ-Ag_3_Li_10_ disordered phase. The simulated source used was CuKα.

4 **-** Ag15Li49-beta-calculated-XRD.txt

X-ray diffraction simulated pattern constituted by two columns with 2θ(°) and Intensity(a.u.) normalized to I_max_ = 100 for the β-Ag_15_Li_49_ phase. The simulated source used was CuKα.

5 **-** Ag4Li9-gamma-disordered-calculated-Cv.txt

Calculated vibrational heat capacity at constant volume for temperatures below the melting point T < 500 K for the γ-Ag_4_Li_9_ disordered phase that was optimized using DFT. The melting point is not known with precision. Two columns with the data: T(K), and Cv(J.K^−1^mol^−1^) included.

6 **-** Ag4Li9-gamma-disordered-calculated-alpha.txt

Calculated thermal linear expansion coefficient for γ-Ag_4_Li_9_ disordered phase for temperatures below the melting point T < 500 K. The melting point is not known with precision. Two columns with the data: T(K), and α(K^−1^) × 10^6^ included.

7 **-** Hf-data-298K.txt

Calculated enthalpies of formation, H_f_, for several phases (stable and unstable) at 298 K. Three columns: compound(stoichiometry), x(Li), and H_f_(kJ.mol of atoms^−1^) included.

8 **-** Gf-data-298K.txt

Calculated Gibbs energies of formation, G_f_, for several phases (stable and unstable) at 298 K. Three columns: compound(stoichiometry), x(Li), and G_f_(kJ.mol of atoms^−1^) included.

9 **-** Hf-data-320K.txt

Calculated enthalpies of formation, H_f_, for several phases (stable and unstable) at 320 K. Three columns: compound(stoichiometry), x(Li), and H_f_(kJ.mol of atoms^−1^) included.

10 **-** Gf-data-320K.txt

Calculated Gibbs energies of formation, G_f_, for several phases (stable and unstable) at 320 K. Three columns: compound(stoichiometry), x(Li), and G_f_(kJ.mol of atoms^−1^) included.

11 **-** Hf-data-425K.txt

Calculated enthalpies of formation, H_f_, for several phases (stable and unstable) at 425 K. Three columns: compound(stoichiometry), x(Li), and H_f_(kJ.mol of atoms^−1^) included.

12 **-** Gf-data-425K.txt

Calculated Gibbs energies of formation, G_f_, for several phases (stable and unstable) at 425 K. Three columns: compound(stoichiometry), x(Li), and G_f_(kJ.mol of atoms^−1^) included.

13 **-** Hf-data-600K.txt

Calculated enthalpies of formation, H_f_, for several phases (stable and unstable) at 600 K. Three columns: compound(stoichiometry), x(Li), and H_f_(kJ.mol of atoms^−1^) included.

14 **-** Gf-data-600K.txt

Calculated Gibbs energies of formation, G_f_, for several phases (stable and unstable) at 600 K. Three columns: compound(stoichiometry), x(Li), and G_f_(kJ.mol of atoms^−1^) included.

14 **–** Table 1

**Table 1:** Details on the phases' structure and optimization.

Compound	x(Li)	Initial structure space group	Method used to obtain the final structure
Ag_15_Li	0.0625	Fm-3m	SQS
Ag_7_Li	0.125	Fm-3m	SQS
Ag_13_Li_3_	0.1875	Fm-3m	SQS
Ag_3_Li	0.25	Fm-3m	SQS
Ag_11_Li_5_	0.3125	Fm-3m	SQS
Ag_17_Li_15_	0.46875	Pm-3m	random substitution
AgLi	0.5	I4_1_/amd	N.A.
AgLi	0.5	Pm-3m	N.A.
Ag_63_Li_65_	0.50781	Pm-3m	random substitution
Ag_31_Li_33_	0.51563	Pm-3m	random substitution
Ag_61_Li_67_	0.52344	Pm-3m	random substitution
Ag_59_Li_69_	0.53906	Pm-3m	random substitution
Ag_55_Li_73_	0.57031	Pm-3m	random substitution
Ag_51_Li_77_	0.60156	Pm-3m	random substitution
Ag_4_Li_9_	0.69231	I-43m	Disordered as close as published [6,7]
Ag_15_Li_37_	0.71154	I-43m	random substitution
Ag_7_Li_19_	0.73077	I-43m	random substitution
Ag_15_Li_49_	0.76563	Pm-3m	random substitution
AgLi_7_	0.875	Fm-3m	SQS

Details on Ag–Li phases' composition, structures and optimization methods (compound, x(Li), initial structure space group, and method to obtain the final optimized structure).

## 2 Experimental design, materials, and methods

The Ag_30_Li_70_ sample was prepared as described in Ref. [1]. The XRD data were obtained from 10 to 90° (2θ) with a Bragg Brentano configuration for polycrystalline samples with a wavelength of λ = 0.1542 nm which is, in fact, an average of two closely spaced peaks (CuKα1 and CuKα2).

The theoretical background in Ref. [1] explains the calculations of the Thermodynamic data included in this database; the theoretical principles were used as implemented in VASP [2], MT [3] and Phonon [4].

Each phase was optimized from a structure that was obtained using random substitution, special quasirandom structure (SQS), or substitutional search, depending on the type of structure (e.g. fcc or bcc). Since SQS's mimics well the local atomic structure of the random alloy, their electronic properties, calculable via first-principles techniques, provide a representation of the electronic structure of the alloy [5]. Table 1 shows the stoichiometry of the compound, the initial space group, and the method used for obtaining the compounds whose thermodynamic data is included in the dataset associated with this work.
